# Diabetes mellitus increases the susceptibility to encephalitozoonosis in mice

**DOI:** 10.1371/journal.pone.0186954

**Published:** 2017-11-01

**Authors:** Aldo Francisco Neto, Paulo Ricardo Dell’Armelina Rocha, Elizabeth Christina Perez, José Guilherme Xavier, Giovani Bravin Peres, Diva Denelle Spadacci-Morena, Anuska Marcelino Alvares-Saraiva, Maria Anete Lallo

**Affiliations:** 1 Programa de Patologia Ambiental e Experimental, Universidade Paulista (UNIP), São Paulo, Brasil; 2 Departmento de Fisiopatologia, Instituto Butantã, São Paulo, Brasil; Universidade de Sao Paulo Instituto de Ciencias Biomedicas, BRAZIL

## Abstract

Microsporidiosis are diseases caused by opportunistic intracellular fungi in immunosuppressed individuals, as well as in transplanted patients, the elderly and children, among others. Diabetes mellitus (DM) is a metabolic disease characterized by hyperglycemia and decreased T cell response, neutrophil function, humoral immunity failure, increasing the susceptibility to infections. Here, we investigated the susceptibility of streptozotocin (STZ)-induced type I diabetic and/or immunosuppressed mice to encephalitozoonosis by *Encephalitozoon cuniculi*. Microscopically, granulomatous hepatitis, interstitial pneumonia and pielonephritis were observed in all infected groups. STZ treatment induced an immunossupressor effect in the populations of B (B-1 and B2) and CD4^+^ T lymphocytes. Moreover, infection decreased CD4^+^ and CD8^+^ T lymphocytes and macrophages of DM mice. Furthermore, infection induced a significant increase of IL-6 and TNF-α cytokine serum levels in DM mice. IFN-γ, the most important cytokine for the resolution of encephalitozoonosis, increased only in infected mice. In addition to the decreased immune response, DM mice were more susceptible to encephalitozoonosis, associated with increased fungal burden, and symptoms. Additionally, cyclophosphamide immunosuppression in DM mice further increased the susceptibility to encephalitozoonosis. Thus, microsporidiosis should be considered in the differential diagnosis of comorbidities in diabetics.

## Introduction

The Filum Microsporidia comprises more than 1.200 species belonging to the kingdom Fungi. Microsporidia are intracelular pathogens, causing microsporidiosis, mostly encephalitozoonosis [1]. In fact, these diseases have been mainly described as opportunistic in humans and other mammals, such as those with HIV/AIDS, which may develop diarrhea and weight loss [1–3]. The main clinical syndromes include: keratoconjuntivitis, pneumonia, enteritis, nephritis, myositis, meningoencephalitis and disseminated infection [3].

Immune response against *Encephalitozoon* sp. is a cooperative result of adaptive and innate immune responses [4]. T cells are critical for protection against *E*. *cuniculi* infection, especially CD8^+^ T cytotoxic lymphocytes [5]. In fact, T CD8^-^/^-^ knockout mice are more susceptible to encephalitozoonosis, with severe clinical signs (ascitis, lethargy and weight loss). On the other hand, CD4^-^/^-^ T mice infected with *E*. *cuniculi* are resistant as well as in control animals without T cell deficiency [6].

*Encephalitozoon* sp. is an opportunist pathogen in immunosuppressed individuals, the elderly and children [7]. Microsporidiosis are not limited to the immunodefficient *status*, since infection by these pathogens have also been described in immunocompetent individuals [4]. A recent study that investigated parasites in stool samples from 100 DM patients found three diagnosed cases of microsporidiosis [8].

DM is a clinical syndrome associated with deficiency in secretion and/or action of insulin. It is considered an emergent health problem of the 21th century, with about 422 million affected people [9]. Besides typical clinical complications, DM decreases the immune response in T cells, neutrophils, and causes humoral immunity changes, increasing the susceptibility to infection and further disease development [10–14].

Some infections are very prevalent in people with diabetes, such as respiratory infections by *Streptococcus pneumonie*, pneumonia due to *Influenza* virus and *Mycobacterium tuberculosis*. Furthermore, urinary infections such as asymptomatic bacteriuria, fungal and emphysematous cystitis, gastritis due to *H*. *pylori*, and oral and esophagic candidiasis have also been described. Additionally, people with diabetes may also develop more severe symptoms and metabolic complications, such as hypoglycemia, ketoacidosis and coma [15–17]. Moreover, DM people have an increased risk in developing skin and mucosal infections, including those caused by *Candida* spp. [18,19].

In this study, we reported that DM mice were more susceptible to encephalitozoonosis with a decrease of B and T lymphocytes associated with lower IFN-γ serum levels. Moreover, the cyclophosphamide (Cy) immunosuppressive treatment in DM mice further increased the susceptibility to this microsporidiosis.

## Materials and methods

### Animals

*Specific pathogen free* (SPF), 8 weeks old, C57BL/6 male mice were obtained from the “Centro de Desenvolvimento de Modelos Experimentais para Biologia e Medicina” (in Portuguese) from Federal University of São Paulo, Brazil. During the experimental period of 35 days, animals were housed at Paulista University Animal Facility and kept in controlled temperature and humidity in microisolators in SPF conditions.

### Ethics statement

All procedures were approved by the ethics committee of UNIP (protocol number 010/16).

### Pathogen cultivation

Spores of *Encephalitozoon cuniculi* genotype I*–*obtained from Waterborne® Inc. (New Orleans, LA, USA)–were cultivated in rabbit kidney cells (RK-13) at “Laboratório de Culturas Celulares—UNIP” of UNIP. RK cells were kept in Eagle´s medium (Cultilab, Campinas, Brazil) supplemented with bovine fetal serum (BFS) (Cultilab, Campinas, Brazil), non-essential amino acids and pyruvate at 10% and penicillin-streptomycin (Sigma-Aldrich, St Louis, USA), and incubated with 5% CO_2_ at 37C. Every seven days, cultures supernatants were collected and centrifuged for 30 minutes at 500 g to obtain spores, that were further kept at 4°C in PBS 1x. Spores of *E*. *cuniculi* were counted in a Neubauer chamber.

### Induction of DM by STZ

Type 1 DM was induced by intra-peritoneal administration of STZ at 50 mg/kg/day, during 5 consecutive days [20, 21]. Ten days after the end of the treatment, 10 μl of blood was collected from the tail of each DM animal. Subsequently, glycemic values were measured with Accu Chek Active glucometer (Roche, Mannheim, Germany). DM condition was determined when the glycemic level was above 250 mg/dl [20]. This was considered day 0 for experimental infection.

### Treatment with Cy

The immunosuppression group was treated with an intra-peritoneal injection of Cy twice per week (75 mg/kg) (Genuxal®, Asta Medica Oncologia, São Paulo, Brazil), starting at day 0 [22,23].

### Experimental infection

Animals were divided in the following experimental groups: mice infected with *E*. *cuniculi (Infected)*; mice treated with Cy and infected with *E*. *cuniculi (Cy-Infected);* DM mice and infected with *E*. *cuniculi (DM-Infected)*, and DM mice treated with Cy and infected with *E*. *cuniculi (DM-Cy-Infected)*. Infection was done by the I.P. route with 1x10^7^ spores of *E*. *cuniculi* at day 0. Uninfected controls were also kept at the same conditions already described: *Uninfected*, *Cy-Uninfected*, *DM-Uninfected* and *DM-Cy-Uninfected*. During all experimental period (35 days) the blood glucose, temperature and weight were measured, in the mornings without previous fasting.

### Necropsy and tissue sampling

At 35 days post infection, all animals were humanely euthanatized with ketamine (50 mg/ml), xylazine (20 mg/ml) and fentanyl (0,05 mg/ml) injected by the I.P. route. About 1 ml of blood was collected by intra-cardiac puncture. Subsequently, cells were obtained from the peritoneal cavity (PerC) by successive washes of the PerC with 2 ml of PBS supplemented with 2% BFS, until the volume of 10 ml. Afterwards, half of the spleen was collected and macerated in a 70 μm cell strainer. Samples of the spleen, liver, kidneys, lungs and duodenum were fixed in 10% buffered formalin solution for 72 hours and routinely processed for histopathology. All tissue samples were colored with HE procedure and evaluated under light microscopy. The hepatic lesions were photographed at x400 magnification using Opticam® photomicroscope. Five images from “hot spots” region were choosing for morphometric analysis. The lesions were manually delineated using the cursor and the area automatically calculated by MetaMorph Software.

### Phenotypical analysis of immune cells

Pellets obtained from the spleen and PerC were centrifuged at 550 g for 5 minutes. Supernatants were discarded and red blood cells were removed with 2 ml of hemolytic buffer at room temperature for 5 minutes. Subsequently, 10 ml of PBS-BFS 2% (Cultilab, Campinas, Brazil) was added and centrifugation was performed at 550 g for 5 minutes. Then, aliquots of samples were counted in a Neubauer chamber. To block Fc receptors, anti-CD16/CD32 antibody was diluted in PBS supplemented with bovine serum albumin (BSA) 1%. Afterwards, cells were washed and further incubated with the following monoclonal antibodies: rat anti-mouse CD19 Peridinin Chlorophyll Protein (PerCP)- conjugated or Allophycocyanin (APC)- conjugated, rat anti-mouse CD23 Phycoeritrin (PE)- conjugated, rat anti-mouse F4/80 APC- conjugated, rat anti-mouse CD11b APC-Cyano Dye 7 (APC-Cy7)- conjugated, rat anti-mouse CD4 PerCP- conjugated and rat anti-mouse CD8 Fluorescein isothiocyanate (FITC)- conjugated (BD-Pharmingen, San Diego, USA). Cell phenotypes were determined as follows: macrophages (CD19^-^CD11b^+^F4/80^+^), B-1 cells (CD23^-^CD19^+^), B-2 cells (CD23^+^CD19^+^), pre B-1 cells-derived phagocyte (Pre B-1 CDP) (CD19^+^CD11b^+^F4/80^+^) [20], CD4^+^ T lymphocytes (CD19^-^CD4^+^) and CD8^+^ T lymphocytes (CD19^-^CD8^+^). After 20 minutes at 4°C, cells were washed and re-suspended in 300 μl of PBS for flow cytometry acquisition. Gates for cell characterization were determined based on patterns of cell size and granularity. Data was acquired with FACSCanto II (BD Biosciences, Mountain View, USA) at UNIFESP (Discipline of Immunology) and the software FlowJo was used for data analysis (FlowJo LLC, Data Analysis Software, Ashland, USA).

### Cytokine quantification

The serum from each animal was collected and stored at −20°C. “CBA Mouse Th1/Th2/Th17 Cytokine Kit” (BD Biosciences, Mountain View, CA, USA) was used according to the manufacturer’s instructions. Briefly, 25 μl of serum from each animal was mixed with each capture bead (IL-2, IL-4, IL-6, IL-10, IL-17, IFN-γ and TNF-α) conjugated with APC and the PE secondary antibody for 2 hours at room temperature under light protection. Subsequently, samples were washed, centrifuged and re-suspended with wash buffer for acquisition with the Flow Cytometer FACSCanto II (BD Biosciences, Mountain View, CA, USA). Analysis was done with the software FCAP Array 3.0. Only the detected cytokines and statistically different results were represented in graphics.

### Fungal burden

To investigate the level of infection in the experimental groups, *E*. *cuniculi* spores were quantified in the PerC wash of infected mice (see PerC processing above). Briefly, 10 μl from PerC suspension were used for spore visualization of counting by using the fluorescent staining Calcofluor (Sigma-Aldrich, St Louis, USA). Fungal burden was determined by randomly selecting and photographing 10 different fields at 400x. Mean of spores counted were represented in graphics.

### Statistical analysis

The normality was calculated with the Shapiro-Wilk test, and the homogeneity of the variance between the groups was verified by the Levene test. Variance analysis (ANOVA) of one and two ways was done, with Tukey post-tests. Confidence intervals were 95% of the media, and analysis was done using the *bootstrap* tool. In all cases, the level of significance was α<0,05. All analyses were done with the software “IBM SPSS *Statistics*” version 21.0 for Windows® (IBM Corporation, Armonk, EUA). GraphPad Prism software (GraphPad Software Inc, La Jolla, USA), version 5.0 for Windows ® or Microsoft Excel were used to build the graphics.

## Results

### Diabetic mice showed more symptoms and increased fungal burden

**Fig 1:**
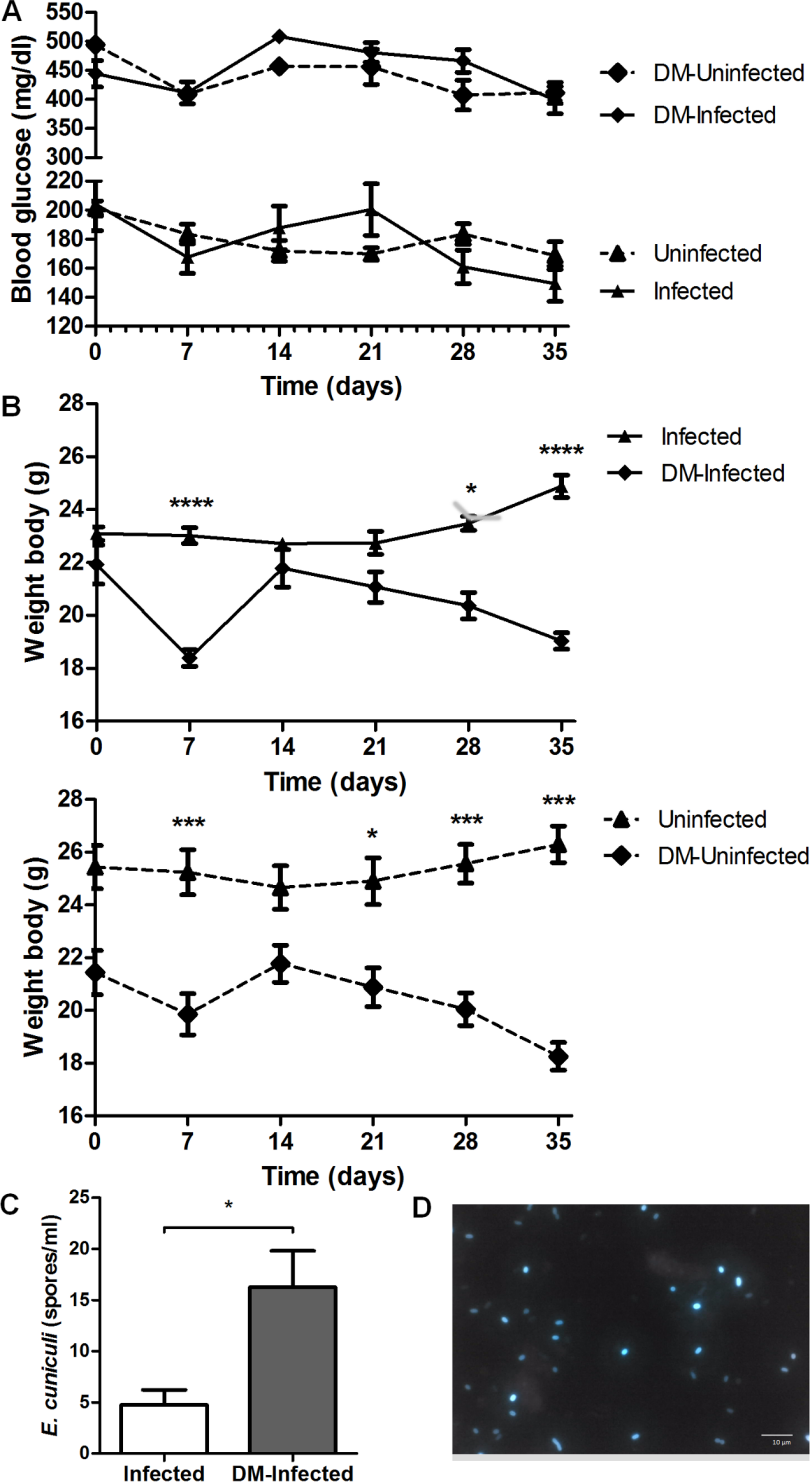
Blood glucose, body weight and fungal burden parameters in mice of experimental groups: *Infected*, *Uninfected*, *DM-Uninfected* and *DM-Infected*. A) Blood glucose variation between groups during all experimental period. B) Body weight variation of *DM-Infected* and *Infected* mice and its respective controls. One way variance analysis (ANOVA) with Tukey post test showed *** p < 0,01. C) Fungal burden in *Infected* and *DM-Infected* mice by *E*. *cuniculi*. Non-parametric *t-test* showing * p<0.01, comparing each group of each period, separately. D) *E*. *cuniculi* spores observed in the PerC of *DM-Infected* mice stained by Calcofluor fluorescent staining.

Experimental infection of diabetic mice was performed to investigate the susceptibility to encephalitozoonosis in chronic metabolic diseases such as DM. The animals treated with STZ that developed diabetes after 10 days of the end of treatment, with blood glucose levels higher then 250 mg/dl were included in this study (Fig 1A). As expected non diabetic mice had normal levels (below to 250 mg/dl). Moreover, the median of body weight in all diabetic (infected and non infected) mice was lower than in non-diabetic mice. Weight loss was more severe by the end of the experiment (Fig 1B).

Serosanguinous exudate was observed only in DM mice and infected with *E*. *cuniculi (DM-Infected*). No symptoms were observed in the other groups. Moreover, a higher fungal burden was observed in *DM-Infected* than infected mice, showing that DM promotes more susceptibility to encephalitozoonosis and disease progression (Fig 1C and 1D).

### DM influences the microscopic lesions associated to *E*. *cuniculi* infection

**Fig 2:**
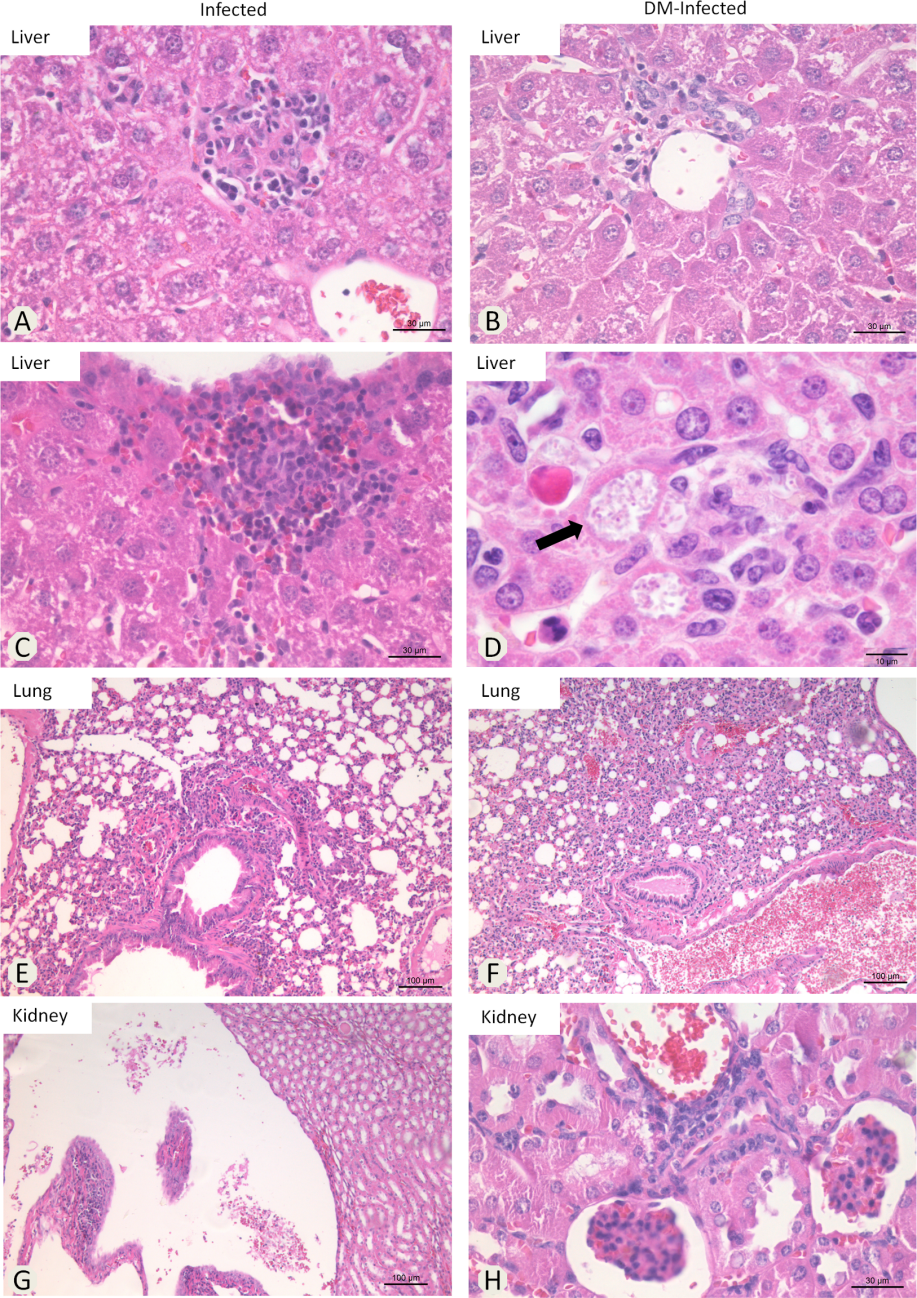
Photomicrographs of histopathological lesions in *E*. *cuniculi Infected* and *DM-Infected* mice. Liver—mononuclear inflammatory infiltrate located at A) parenchyma, B) portal vein, C) under capsule and D) *E*. *cuniculi* clusters into inflammatory infiltrate. Lungs–E and F) interstitial pneumonia. Kidney–G) pyelonephritis and H) nephritis (HE).

Granulomatous multifocal hepatitis was observed randomly in all *E*. *cuniculi* infected groups (*DM-Infected* and *Infected*) (Fig 2A, 2B and 2C). Spores clusters were observed associated or not with inflammatory infiltrates (Fig 2D) in *DM-Infected* mice. Interstitial multifocal pneumonia was observed in all *Infected* groups (Fig 2E); these lesions were more severe in *DM-Infected* animals (Fig 2F). Renal lesions included pielonephritis (Fig 2G) and multifocal interstitial nephritis (Fig 2H). Lesions into the liver were measured in all infected groups. The statistical analysis did not demonstrate significant differences between the areas calculated (Table 1). However, we observed more images containing granulomatous infiltrate in *Infected* mice compared with the *DM-Infected* group. No lesions were observed in *Uninfected* mice.

**Table 1:** Morphometric analyses of hepatic lesions from *E*. *cuniculi* infected mice. Lesions´ area of the liver from animals treated or not with cyclophosphamide (Cy) and treated or not with STZ to development of Type 1 Diabetes mellitus (DM).

group/hepatic lesion	area *	standard deviation*
Infected	1.230	.300
Cy-infected	.746	.197
DM-infected	1.017	.775
Cy-DM-infected	1.183	.888

*pixels, x10^6^; No statistical difference were observed between the groups (ANOVA/Tukey-Kramer).

### DM decreased immune cells population in the peritoneum and spleen

**Fig 3:**
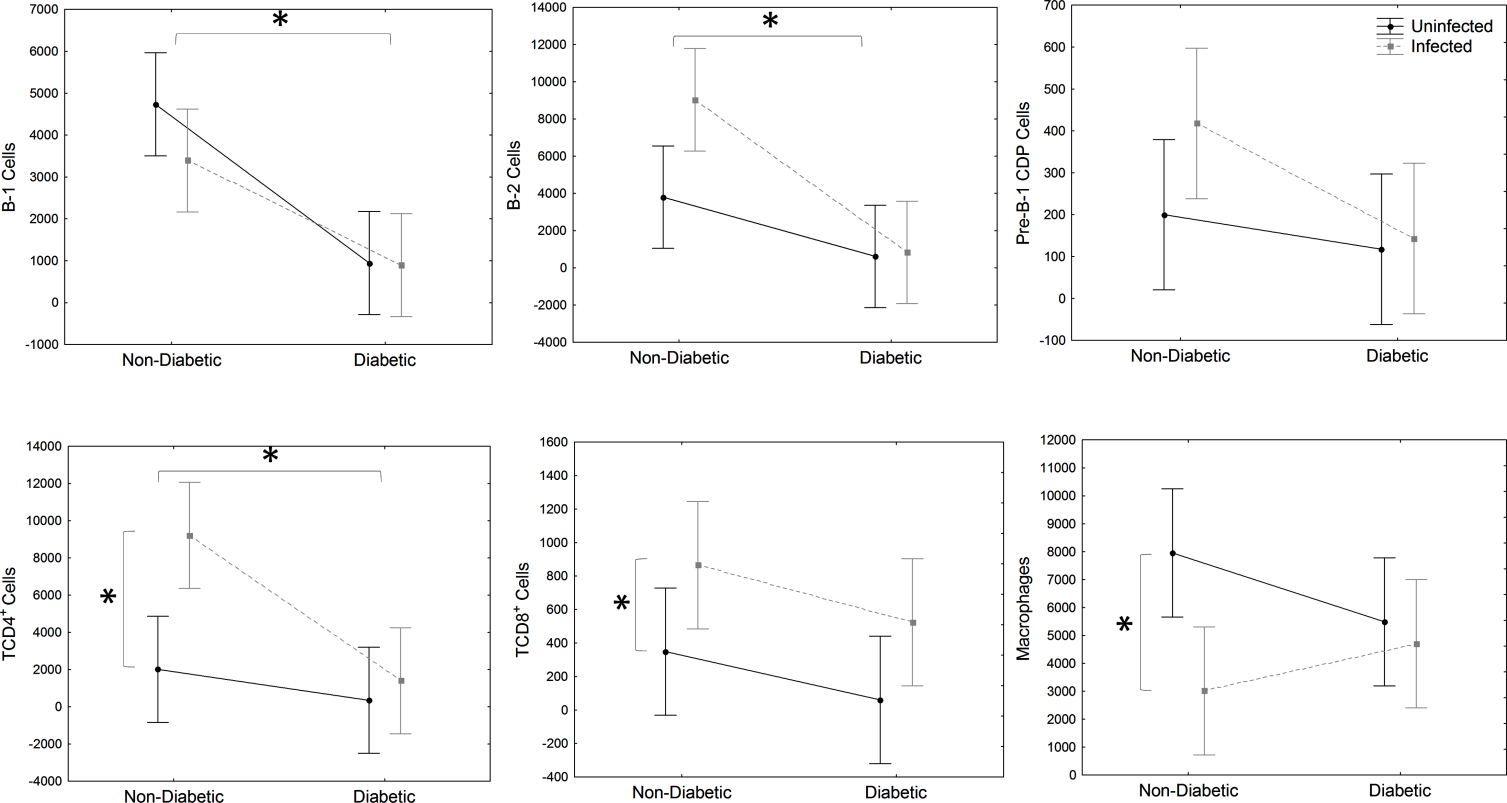
Peritoneal immune cell analysis. Evaluation of B-1 (CD23^-^CD19^+^), B-2 (CD23^+^CD19^+^), Pre-B-1CDP (CD19^+^CD11b^+^F4/80^+^) cells, CD4^+^ (CD19^-^CD8^-^CD4+), CD8^+^ (CD19^-^CD4^-^CD8^+^) T lymphocytes and macrophages (CD19^-^F4/80^+^CD11b^+^) from PerC of STZ-induced DM mice infected with *E*. *cuniculi* compared with its controls. Two ways variance analysis (ANOVA) revealed * p<0,05.

Phenotypical cell analysis included the quantification of macrophages, B and T lymphocytes. Pre-B-1 CDP is a stage of differentiation of B-1 in B-1 CDP, which is described in infection and also diabetes mice model [20,21]. There was a significant decrease of peritoneal B-1, B-2 and CD4^+^ T lymphocytes of *DM* and *DM-Infected* mice than non-diabetic (*Infected* and *Uninfected*) mice (Fig 3), showing that DM condition decreased the immune response.

Moreover, when comparing *Infected* and *DM-Infected* groups with its controls (*Uninfected* and *DM-Uninfected*), CD4^+^ T and CD8^+^ T cells increased (Fig 3). However, macrophages decreased significantly in *Infected* and *DM-Infected* compared to the other groups (Fig 3). There was no significant change regarding B-1, Pre-B-1 CDP and B-2 cells in infection.

These data suggest a possible immunosuppressive effect of diabetic status in B and T lymphocytes in the peritoneal cavity, which may have increased the susceptibility to encephalitozoonosis.

**Fig 4:**
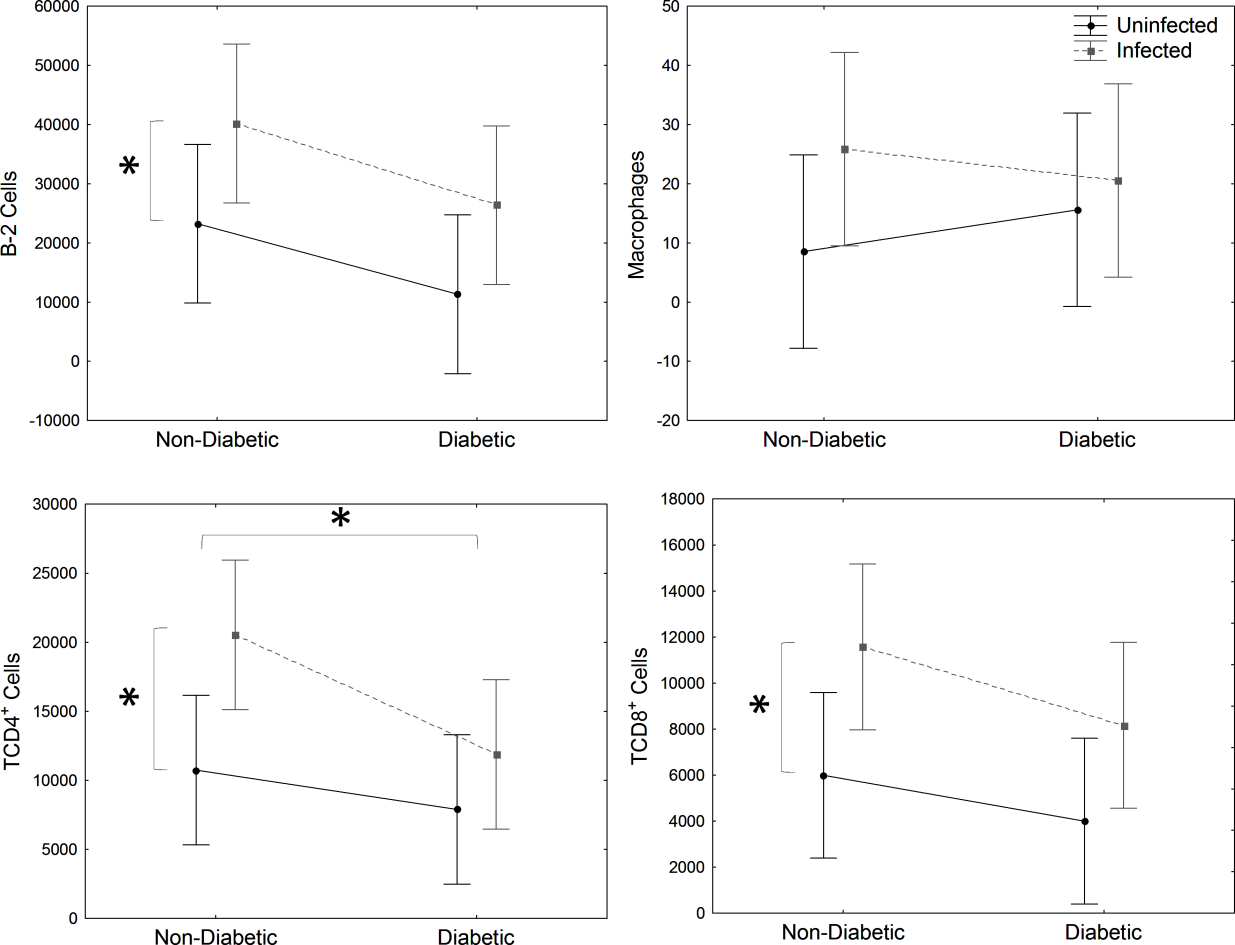
Spleen immune cell analysis. Evaluation of B-2 (CD23^+^CD19^+^) cells, CD4^+^ (CD19^-^CD8^-^CD4^+^) and CD8^+^ T lymphocytes (CD19^-^CD4^-^CD8^+^) and macrophages (CD19^-^F4/80^+^CD11b^+^) from spleen of STZ-induced DM mice infected with *E*. *cuniculi* compared with its controls. Two ways variance (ANOVA) analysis revealed p<0,05*.

In the spleen, a higher quantity of B-2, CD4^+^ and CD8^+^ T cells was observed in infected mice (*Infected* and *DM-*infected) than uninfected mice (*Uninfected* and *DM-Uninfected*) (Fig 4). However, CD4^+^ T cells decrease in *DM* and *DM-Uninfected* mice (Fig 4). There was no difference in macrophages population.

### DM *E*. *cuniculi* infected mice showed important decrease in IFN-γ levels

**Fig 5:**
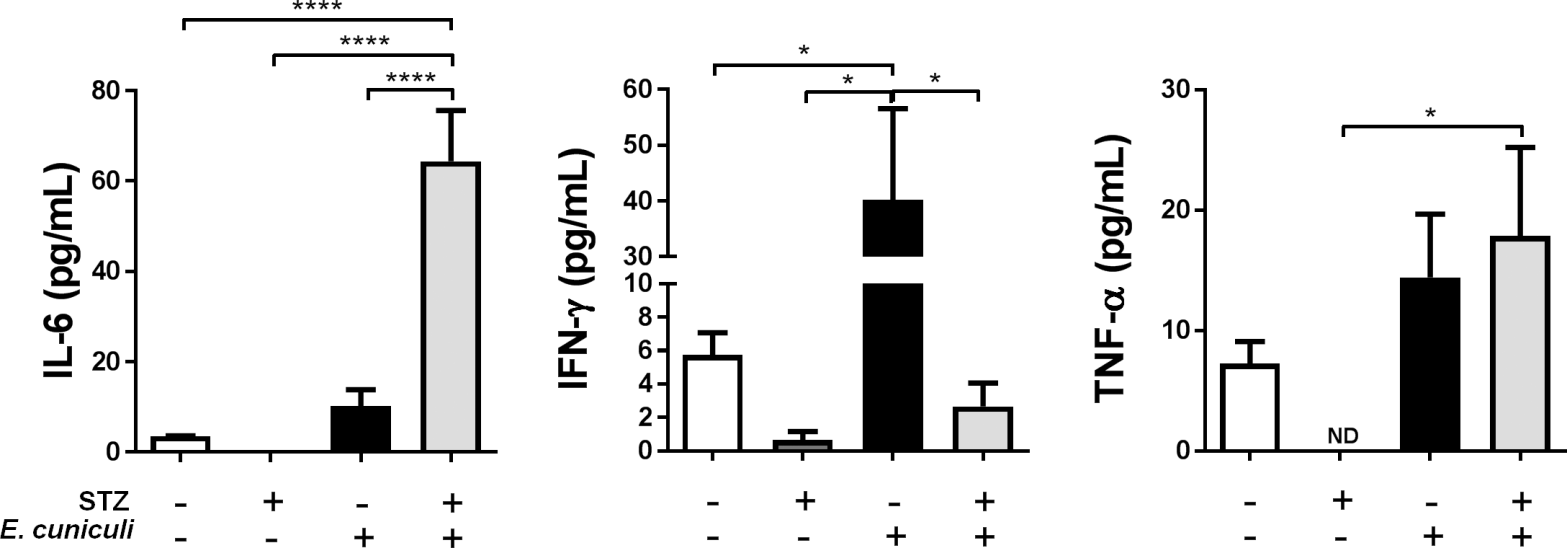
IL-6, IFN-γ and TNF-α cytokines levels in the serum of STZ-induced DM mice infected with *E*. *cuniculi* compared with its controls mice. + and–means presence and absence, respectively, of STZ treatment and *E*. *cuniculi* infection. Two ways variance analysis (ANOVA) revealed * p<0,05 and **** p<0,0001.

*E*. *cuniculi* infection significantly increased pro-inflammatory *Th1* cytokines, mainly IFN-γ in *Infected* group (Fig 5), which is the most important cytokine for the resolution of encephalitozoonosis. In *DM-Infected* mice IFN- γ levels were decreased and IL-6 and TNF-α were increased (Fig 5).

### Cy immunosuppression becomes *DM-Infected* mice more sick

**Fig 6:**
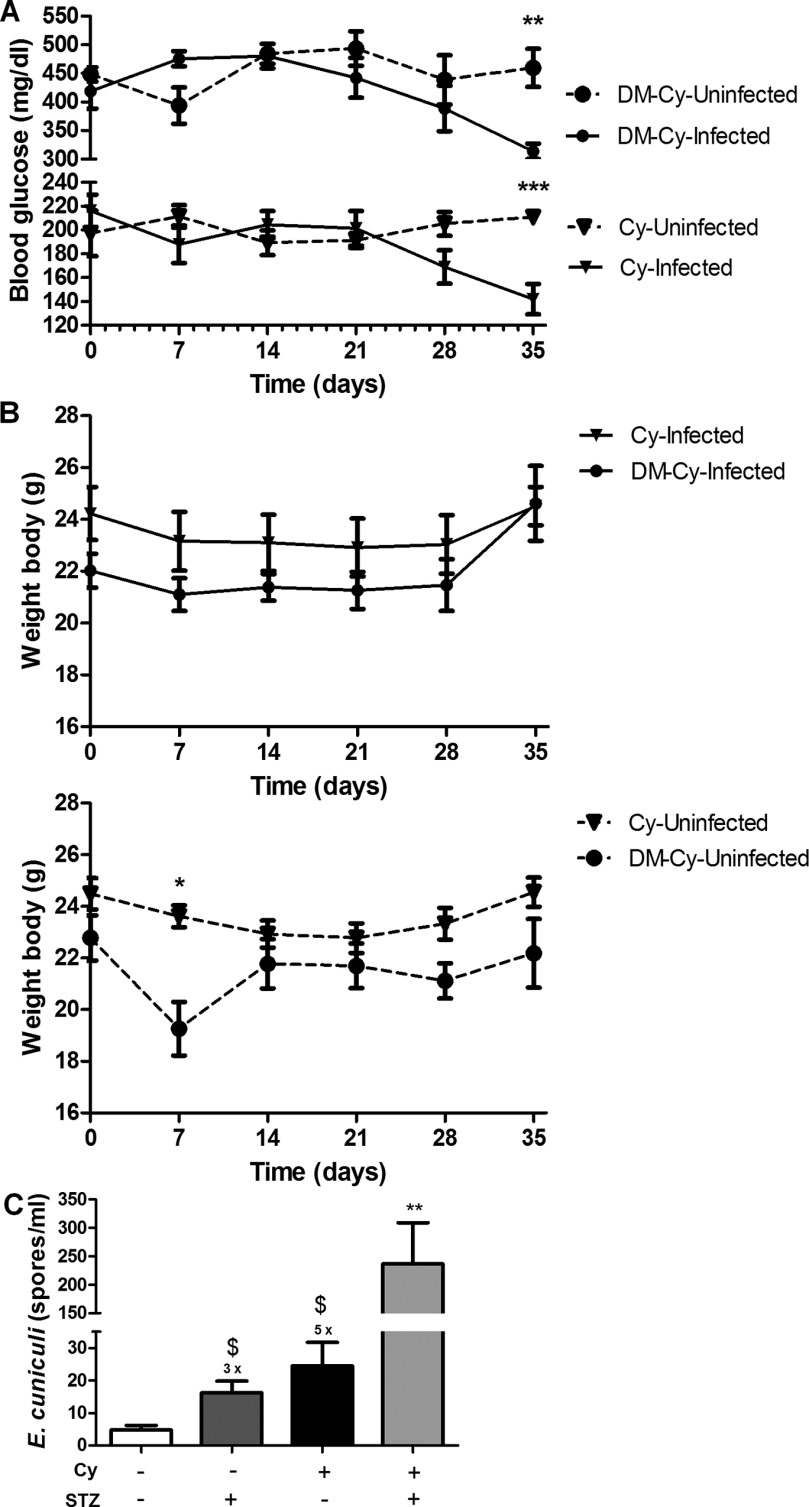
Blood glucose, body weight and fungal burden parameters in mice immunosuppressed with cyclophosphamide (Cy): *Cy-Infected*, *Cy-Uninfected*, *DM-Cy-Uninfected* and *DM-Cy-Infected*. A) Blood glucose variation between immunosuppressed groups during all experimental period. B) Body weight variation of *DM-Cy-Infected* and Cy-*Infected* mice and its respective controls. C) Fungal burden in the PerC of *E*. *cuniculi* infected mice. + and–means presence or absence, respectively, of Cy treatment and *E*. *cuniculi* infection x means time folds compared to *Uninfected* (-/-) group. Non parametric *t-test* showed * p<0,05 and ** p<0,01 (A e B) and One way variance analysis (ANOVA) revealed $ p<0,05 comparing with uninfected groups and ** p<0,01 compared to the other groups.

Immunosuppression may increase the susceptibility to encephalitozoonosis in both humans and animals. Thus, we further investigated the effects of immunosuppression by Cy in DM mice infected with *E*. *cuniculi* (*DM-Cy-Infected*). The blood glucose levels were above 400 mg/dl during the experimental period, except at 35 DPI, when it decreased (313 mg/dl), especially compared to *DM-Cy-Uninfected* mice (459 mg/dl) (Fig 6). The same was observed in non-diabetic mice, since the blood glucose mean levels of *Cy-Infected* were lower (142 mg/dl) than *Cy-Uninfected* (210 mg/dl) (Fig 6A). These data suggest that encephalitozoonosis associated with Cy immunosuppression caused a metabolic change decreasing the blood glucose levels of DM mice. On the other hand, *DM-Cy-Infected* mice were sicker than other groups Cy or non-Cy, as observed in the parameters described below. The body weight not changed in Cy immunosuppressed mice both DM and non-DM (Fig 6B), probably because this mice showed more pronounced ascites.

*Cy-Infected* mice showed a serosanguinous exsudate and splenomegaly. However, a large abdominal distention with abundant serosanguinous exsudate, lethargy and apathy were observed in *DM-Cy-Infected* mice. Although deaths were not observed, animals were euthanized due to poor clinical conditions in *DM-Cy-Infected* group at 35 DPI. No clinical symptoms were observed in *Uninfected* controls. A higher fungal burden (5-folds) was observed in *Cy-Infected* mice compared to *Infected* mice (Fig 6C). Moreover, *DM-Cy-Infected* had a 30-folds higher fungal burden than *Infected* mice (Fig 6C), showing that immunosuppression plus DM condition increased the susceptibility to encephalitozoonosis development. Histopathological hepatic lesions in *DM-Cy-infected* mice were similar to *DM-infected* mice (Table 1).

Cy treatment significantly decreased B-1, B-2, pre-B-1 CDP, CD4^+^ and CD8^+^ T cells in the peritoneum and spleen of Cy-*Uninfected* mice compared to *Uninfected* control, showing a lymphocytic immunosuppressive effect (p<0,05). Only splenic macrophages increased due to Cy treatment (p<0,05).

**Fig 7:**
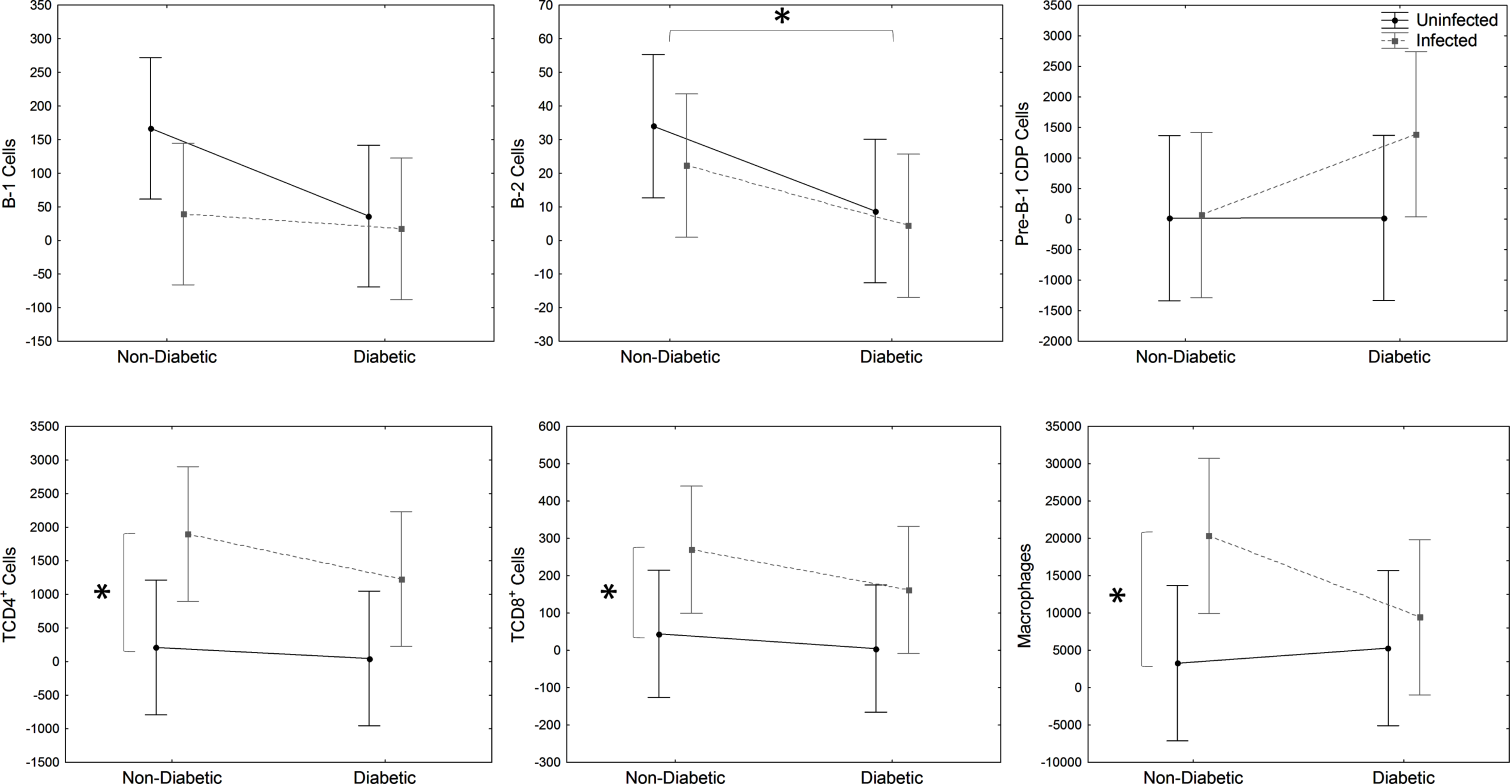
Peritoneal immune cell analysis. Evaluation of B-1 (CD23^-^CD19^+^), B-2 (CD23^+^CD19^+^), Pre-B-1CDP (CD19^+^F4/80^+^CD11b^+^) cells, CD4^+^ (CD19^-^CD8^-^CD4+) and CD8^+^ (CD19^-^CD4^-^CD8^+^) T lymphocytes and macrophages (CD19^-^F4/80^+^CD11b^+^) from PerC of Cy immunosuppressed and STZ-induced DM mice infected with *E*. *cuniculi* compared with its controls. Two ways variance analysis (ANOVA) revealed * p<0,05.

**Fig 8:**
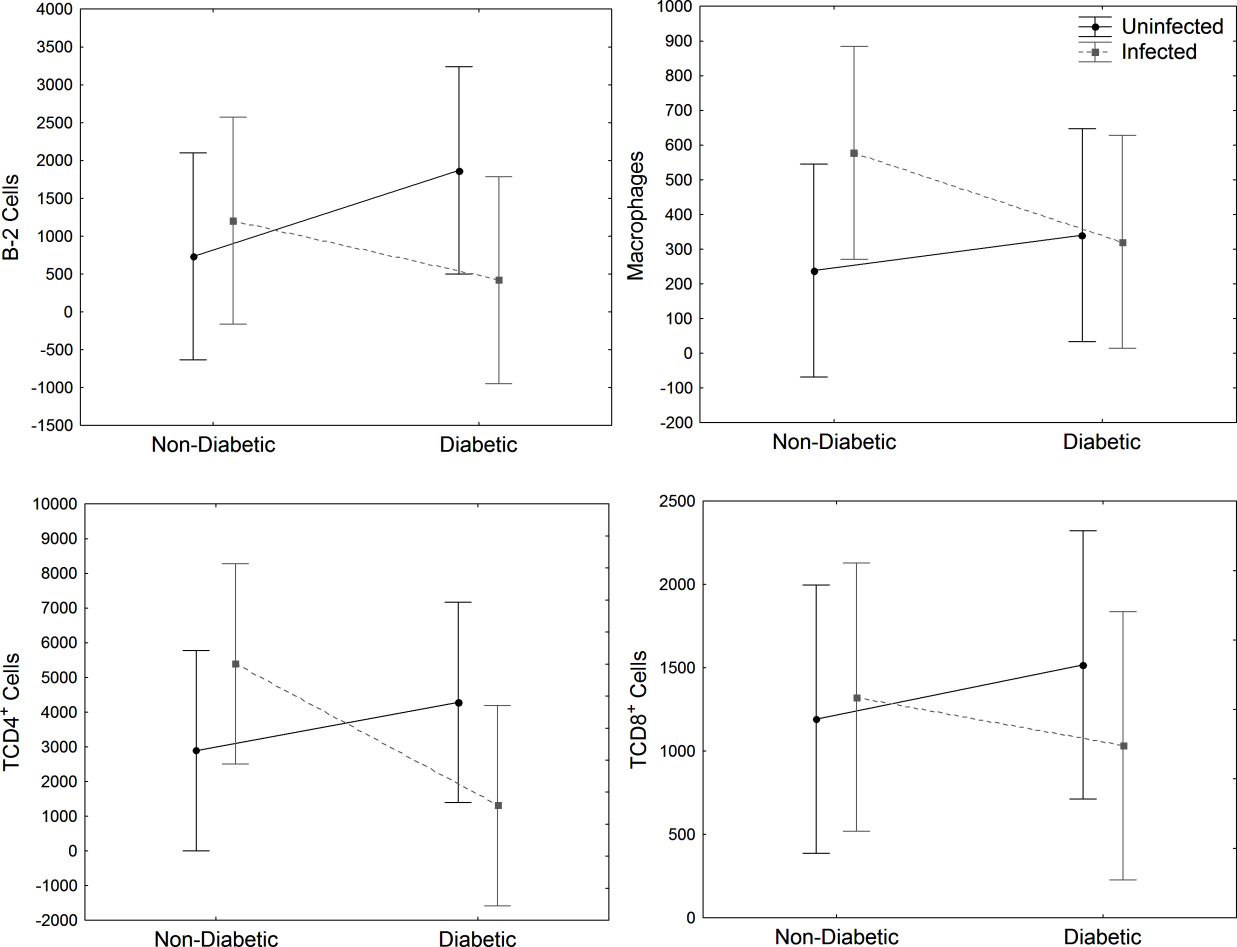
Spleen immune cell analysis. Evaluation of B-2 (CD23^+^CD19^+^) cells, CD4^+^ (CD19^-^CD8^-^CD4+), and CD8^+^ (CD19^-^CD4^-^CD8^+^) T lymphocytes and macrophages (CD19^-^F4/80^+^CD11b^+^) in spleen Cy immunosuppressed and STZ-induced DM mice infected with *E*. *cuniculi* compared with its controls. Two ways variance analysis (ANOVA) revealed * p<0,05.

Peritoneal B-2 cells decreased significantly in *DM-Cy-Infected and DM-Cy-Uninfected* compared to non-DM groups (*Cy-Infected* e *Cy-Uninfected*) (Fig 7). Although the population of immune cells in the peritoneum decreased by Cy treatment, *E*. *cuniculi* infection increased CD4^+^ and CD8^+^ T cells and also macrophages in *Cy-Infected* and *DM-Cy-Infected* mice compared to uninfected controls (*Cy-Uninfected* and *DM-Cy-Uninfected*), showing the importance of these cells against microsporidiosis, even in an immunosuppressive condition (Fig 7). There are no differences pointed in the spleen cells population analyzed (Fig 8).

**Fig 9:**
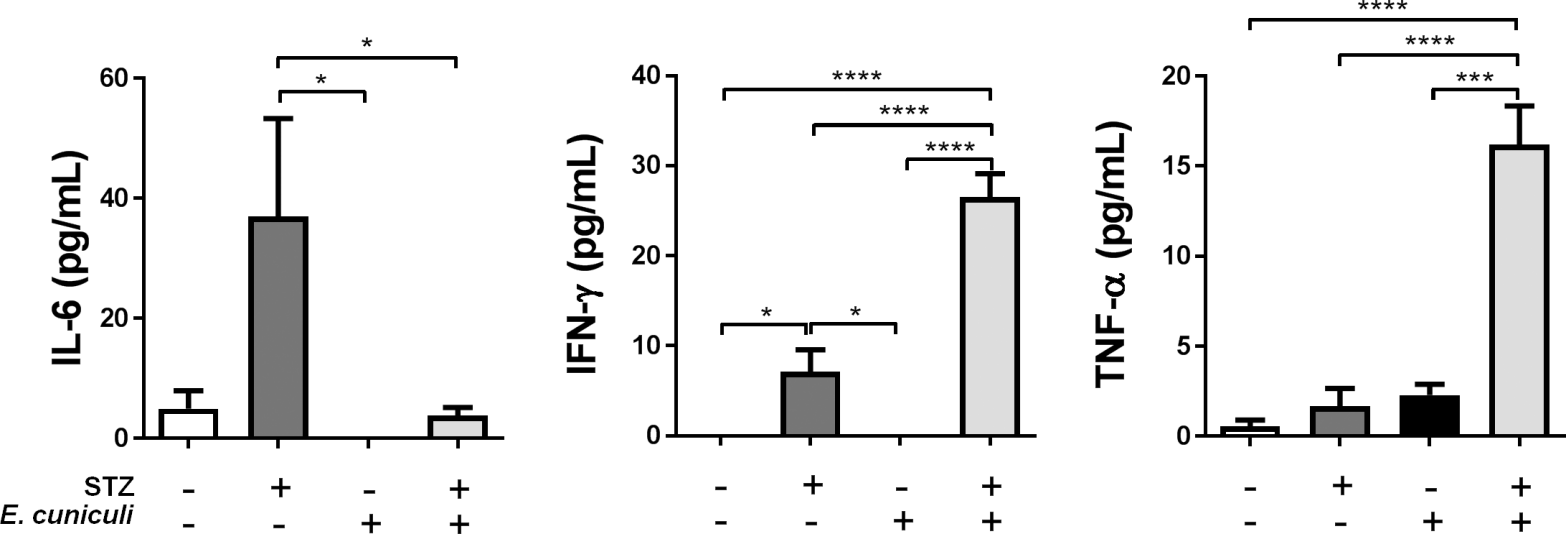
IL-6, IFN-γ and TNF-α cytokines levels in the serum of Cy immunosuppressed mice and STZ-induced DM mice infected with *E*. *cuniculi* compared with its controls. + and–means presence and absence, respectively, of STZ treatment and *E*. *cuniculi* infection. One way variance analysis (ANOVA) with Tukey *posttest* showed * p<0,05, *** p<0,001 and **** p<0,0001.

*E*. *cuniculi* infection increased IFN-γ and TNF-α serum levels in *Cy-DM-Infected* animals compared to the other groups (Fig 9). Additionally, IL-6 and IFN- γ levels increased in *DM-Uninfected* mice (Fig 9).

## Discussion

DM is a clinical pathological condition that cause functional abnormalities in patients, such as insufficient immune response against bacterial and mycotic infections [12–15]. Many immunological alterations are related to hipoinsulinemia and hipoglycemia levels in the blood, altering the function of lymphoid organs [10]. For instance, the thymus of DM individuals showed atrophy, loss of cortical-medullar function, increased of extracellular matrix and decreased expression of CCL25 and CXCL12 chemokines [24]. These changes are transient and its detailed clinical importance in DM people has not been fully described. In the present study, DM was induced by STZ in mice that were infected with *E*. *cuniculi*. Results showed a higher fungal burden, clinical changes and microscopic lesions in the lung in *DM-Infected* mice compared to *Infected* mice. These data demonstrated for the first time that type 1 DM increased the susceptibility to encephalitozoonosis.

HIV-positive, chemotherapy immunosuppressed and diabetes patients, pregnant women, the elderly and children are the mainly risk group to infections by opportunistic pathogens [7,25–27]. So far, the only etiopathological relationship between Microsporidia and Diabetes was a case report of cerebral abscess co-infection due to *Encephalitozoon cuniculi* genotype I and *Streptococcus intermedius* in a type II DM patient without immunosuppression [28]. Together, the present results suggest that encephalitozoonosis should be considered an opportunistic disease in DM patients.

The main pathogenic mechanisms associated with the susceptibility of DM individuals to infectious diseases include: the increased virulence due to a hyperglycemia and decreased chemotaxis (interleukins production, leukocyte mobilization and phagocytic activity against infection), glycosuria and intestinal and urinary dysmotility [29].

In DM, innate immune response against infection has been more studied than adaptive immune response. Mice with type 1 DM (STZ-induced) and type 2 DM (due to obesity) infected with *Staphylococcus aureus* had a more severe disease than its controls, which was related to a decrease in humoral response [30]. Moreover, the increase in disease development in DM individuals has also been associated with a decrease of T cell immune response [18,19]. In our study, B-1, B-2 and CD4^+^ T cells decreased significantly in the peritoneum of *DM-Uninfected* and *DM-Infected* mice compared to non-DM mice (*Uninfected* and *Infected*). Previous studies showed a protective effect against encephalitozoonosis mediated by CD8^+^ T lymphocytes [5,31]. Corroborating these results, we showed increased CD8^+^ T lymphocytes in the PerC and spleen of *DM-Infected* and *Infected* groups. Moreover, previous investigations from our group already showed the importance of B and T lymphocytes in the acquired immunity against encephalitozoonosis [32]. Herein, we suggest that the decrease of B and T populations caused by DM condition may have resulted in increased susceptibility of the encephatozoonosis. Thus, although CD8^+^ T cells are mainly population against encephalitozoonosis, the decrease of B-1 cells reinforces the importance of this population against this intracellular pathogen.

The effect of glucose against the bacteria *Pseudomonas aeruginosa in vivo* was already shown in mice with hyperglycemia induced by STZ. Bacterial burden was higher in hyperglycemic animals than its controls and also the metformin treatment reduced both airways glucose and bacterial burden. These data suggest that glucose in the airways is a critical determinant for increased bacterial burden in DM animals [33]. Considering that *E*. *cuniculi* is an intracellular pathogen, we speculate that the hyperglycemia could facilitate the microsporidia surveillance in DM mice by in a different way.

In a previous study, we showed that BALB/c mice were more resistant against encephalitozoonosis than BALB/c XID mice, partly due to B-1 cells [32]. Although B-2 cells may not play a crucial role against encephalitozoonosis, antibody levels in infected animals are very high [2], suggesting B-2 cells role in acquired immunity. Therefore, the present results suggested that B-1 and B-2 cells decreased in DM mice could contribute to the increase of encephalitozoonosis susceptibility.

Both *in vitro* and *in vivo* studies showed that *Th1* inflammatory cytokines (mainly IFN-γ) are crucial against *E*. *cuniculi* infection, activating both cytotoxic CD8^+^ T lymphocytes and macrophages [34,35]. In our study, *Infected* animals showed a predominant *Th1* cytokine response (higher levels of IFN-γ and TNF-α). Th1 cytokines (mainly IFN-γ) develop an important role during microsporidia infection in immunodeficient mice [36], corroborating our results. IFN-γ^-/-^ *knockout* mice are unable to solving *E*. *cuniculi* infection [37] and the adoptively transfer of splenic cells increase their surveillance, suggesting that NK cells producing IFN-γ are important to *E*. *cuniculi* destruction [38].

Mononuclear cells from DM people secrete more IL-1 and IL-6 when treated with lipopolysaccharide (LPS) [13]. In the present results, DM-*Infected* and *DM-Cy-Uninfected* mice showed a higher level in IL-6, suggesting that DM may also modulate a pro-inflammatory state of the organism. However, other studies reported the decrease in interleukin secretion as a consequence of intrinsic defects in the cells of DM patients [11,39]. The increased glycation in DM individuals may also inhibit IL-10 production by myeloid cells, and IFN-γ /TNF-α by T lymphocytes [40]. The low IL-10 levels may block anti-inflammatory effects on macrophages in DM condition. Also, high levels of IL-6 and IL-12 founded in DM NOD mice address to Th1 pro-inflammatory profile, allowing macrophages precursors in hyperglycemic environment to target to Th1 response even before to entry in infected/inflamed tissue [41]. In our results, DM condition also decreases IFN-γ levels and *E*. *cuniculi* infection increases this cytokine in non-DM mice. We speculate that the decrease in IFN-γ in DM mice associated with the decrease in PerC lymphocytes could increase disease susceptibility.

Moreover, Cy treatment in *DM-Cy-Infected* animals may have caused an immunomodulatory effect, since IFN-γ increased in both *DM-Cy-Infected* and *DM-Cy-Uninfected*. Even though, CD8^+^ T cells decreased significantly in *DM-Cy-Infected* mice, which was sickest of all infected groups. A higher mortality rate was observed in mice treated with STZ after intra-tracheal *Klebsiella pneumonia* infection [14]. There was a decrease in granulocyte chemotaxis to the alveolar space associated with a decreased of chemokines (CXCL1, CXCL2) and cytokines (IL-1β and TNF-α). This result suggests a failure in recruiting granulocytes to the infection, increasing the susceptibility to *K*. *pneumoniae* pneumonia [14]. The present results showed an increase of TNF-α in *DM-Infected* mice, however, as described above, this cytokine is not the most effective against encephalitozoonosis.

In previous investigations, we showed that mice immunosuppressed with drugs are important biological models for the study of murine encephalitozoonosis, since immunosuppression favors disease progression, mimicking natural conditions of immunosuppression [22,23,42]. Cy is a cytotoxic alkylating agent, causing cell apoptosis, delaying and suppressing lymphocytic immune response, causing myelosuppression, decreasing neutrophils, T and B lymphocytes, erythrocytes and platelets [43]. In our study, B and T cells decreased significantly by Cy treatment, which was associated with higher fungal burden than infected non-Cy mice, as previously reported. Additionally, Cy treatment may allow depletion of B and T lymphocytes, following extensive mobilization of bone marrow, with cell recovery and proliferation [44,45]. In our study, *E*. *cuniculi* infection in Cy-treated mice caused CD4^+^, CD8^+^ T cells and macrophage increase, which may be due to this secondary bone marrow stimulatory effect together with infection. However, the number of these cells was lower than *Infected* mice, which may further explain why Cy increases disease susceptibility.

We observed a metabolic change probably caused by the association of encephalitozoonosis and Cy treatment, carrying on to a blood glucose decrease of both DM and non-DM mice. Microsporidia influences the biochemistry environmental of the host with energetic costs to fungal. Microsporidia uses ATP, since *E*. *cuniculi* are frequently observed surrounded by mitochondrias in the cytoplasm of parasitized cells [46]. Even though Microsporidia have reminiscent mitochondria (or mitosome), they need to parasitize a cell to replicate. In fact, there are four ATP transporters in microsporidia. One of them is located in mitosome membrane. The ATP produced by host cell is transported from mitochondria to microsporidia cytoplasm or mitosome [47]. Furthermore, it has been reported that microsporidia infection decrease glycogen of the host and also a rapid glucose absorption [48]. These events could be promoted by parasite’s hexokinase into host cells. The hexokinase catalyzes the first step in glycolysis, through the pentose phosphate via. Therefore, the activity of hexokinase of microsporidia inside host cell could increase the synthesis of nucleotides, amino acids and lipids, which are needed for parasite development [49]. We suggest that the metabolic changes due to *E*. *cuniculi* infection could be favored by Cy treatment, promoting susceptibility to disease. This hypothesis is under investigation.

In conclusion, the present results showed that STZ-induced DM mice were more susceptible to encephalitozoonosis, probably due to a decrease in B-1, B-2 and CD4^+^ T cells and decreased IFN-γ levels, consistent with immunosuppressive condition. Overall, *E*. *cuniculi* infection caused more severe disease in DM and Cy-treated mice.
